# Optical Interrogation Techniques for Nanophotonic Biochemical Sensors

**DOI:** 10.3390/s19194287

**Published:** 2019-10-03

**Authors:** Filiz Yesilkoy

**Affiliations:** Department of Biomedical Engineering, University of Wisconsin–Madison, Madison, WI 53706, USA; filiz.yesilkoy@wisc.edu

**Keywords:** nanophotonic devices, plasmonic sensors, all-dielectric metasurfaces, label-free biochemical sensors, spectral interrogation, imaging spectroscopy, intensity interrogation, hyperspectral imaging, phase interrogation, spectrometer-less optics

## Abstract

The manipulation of light via nanoengineered surfaces has excited the optical community in the past few decades. Among the many applications enabled by nanophotonic devices, sensing has stood out due to their capability of identifying miniscule refractive index changes. In particular, when free-space propagating light effectively couples into subwavelength volumes created by nanostructures, the strongly-localized near-fields can enhance light’s interaction with matter at the nanoscale. As a result, nanophotonic sensors can non-destructively detect chemical species in real-time without the need of exogenous labels. The impact of such nanophotonic devices on biochemical sensor development became evident as the ever-growing research efforts in the field started addressing many critical needs in biomedical sciences, such as low-cost analytical platforms, simple quantitative bioassays, time-resolved sensing, rapid and multiplexed detection, single-molecule analytics, among others. In this review, the optical transduction methods used to interrogate optical resonances of nanophotonic sensors will be highlighted. Specifically, the optical methodologies used thus far will be evaluated based on their capability of addressing key requirements of the future sensor technologies, including miniaturization, multiplexing, spatial and temporal resolution, cost and sensitivity.

## 1. Introduction

From early-stage fundamental research to everyday clinical practice, sensors that can specifically and precisely quantify analytes from biosamples are essential analytical tools in biomedical sciences. In medicine, for example, the importance of biomarker profiling for prognostics and diagnostics is rapidly growing with the substantial discoveries of disease-indicating biomarkers, which can also provide novel insights into disease stages [1]. Moreover, targeted molecular therapies are emerging as effective personalized treatments, especially for cancer, where tailored molecules interfere with specific biochemical events influencing the disease progression [2]. Currently, both diagnostic and therapeutic investigations rely on conventional gold-standard analytical techniques, such as mass spectrometry, chromatography and enzyme-linked immunosorbent assay (ELISA). These methods are either destructive or label-based; they require bulky and expensive equipment as well as lengthy and laborious protocols that must be run by trained experts in equipped facilities, such as clinical biochemistry laboratories. Current trends in medicine, including personalized treatments and at-home health monitoring, require biosensing tools that are ubiquitous. Moreover, in order for medicine to benefit from the big data revolution, low-cost and decentralized biosensor networks that can rapidly measure multiple analytes from small sample volumes are needed [3]. Having access to a plethora of biomarker information collected using such sensor networks from large populations can enable the statistical evaluation of therapeutics and drugs, the study of disease models and heterogeneities over patient groups, and the enhancement of medical knowledge through correlations drawn from the big-health data. Another significant impact of these sensors would be on public health, as smart data processing algorithms can track patterns and trends to identify health threats, such as pandemic outbreaks, chemical pollutant exposures, and terrorist attacks, by correlating the health data within a given geographical zone [4,5].

Optical sensors are receiving growing attention as they are strong candidates to become fundamental nodes in future sensor networks. Prominent label-free optical sensor technologies include waveguide-based devices [6], whispering-gallery mode sensors [7], photonic crystals [8] and nanophotonic devices. The development of optical sensors accelerated with the nanotechnology revolution, which brought forward the flourishing field of nanophotonics. Light’s unprecedented interaction with nanostructured materials caused a leap in novel sensor designs and enhanced the naturally weak signals measured with fundamental spectroscopic techniques, such as Raman [9], mid-infrared absorption [10] and terahertz time-domain [11]. In particular, the focusing of free-space-propagating light into nanoscale surface volumes by engineered nanomaterials allowed for the efficient interaction of light with small molecules and, thus, their direct non-destructive detection. Nanoplasmonic sensors, i.e. noble metal nanoparticles (NP) or nanoapertures, gap-separated NP assemblies and periodic NP or nanoaperture arrays forming metasurfaces, are prominent optical sensors that have been at the center of an active research field in the past few decades. These photonic devices exhibit sharp spectral absorption and scattering profiles when the free-space propagating electromagnetic waves excite free electrons of the metal particle creating collective oscillations, famously known as localized surface plasmon resonance (LSPR) [12]. More recently, nanostructured dielectric metasurfaces have emerged as high-performing sensors, addressing the shortcomings of their plasmonic counterparts, offering complementary metal oxide semiconductor (CMOS) manufacturing compatibility and high quality (high-Q) resonances due to low material losses. Commonly in these nanophotonic sensors, nanoresonators efficiently enhance the far-field coupled light into near-field hot spots and create optical resonances at specific wavelengths, which depend on the material, geometry, and arrangement of nanostructures, as well as the optical properties of the sensor’s surrounding media. Analyte binding on these hotspots create local variations in the refractive index (RI), leading to changes in the optical resonance properties of the nanophotonic sensors, which are converted into detection signals through the use of various optical interrogation mechanisms.

Nanophotonic biomolecule detecting systems are promising because they address the key technological challenges of the future sensors. First, they enable direct detection of analytes from liquid samples without the need of exogenous labels. This is a pivotal property in a biosensor design because it facilitates the bioassay procedures by eliminating tedious washing, amplifying, and labelling steps. Since rapid detection is a critical need for many applications, the most prominent benefit of simple analytical measurement protocols is short turnaround time from sample submission to results. As the complexity of a bioassay decreases, the dependence on well-equipped laboratories, wet-bench processes and trained personnel is reduced, opening the way for field-deployable analytical platforms. Moreover, contrary to the labelled assays, direct analyte detection is compatible with real-time continuous data acquisition. This is an essential property to study kinetics and affinity of target-receptor molecule binding events, and to monitor dynamically changing biological systems, such as cell/tissue secretion analysis. Second, the nanophotonic resonators can be excited with free-space propagating light in the visible (VIS) and near infrared (NIR) spectral range and are compatible with far-field readout optics. This is particularly advantageous as the light sources, detectors, and optical components are abundant in this spectral range. Furthermore, the resonances in VIS-NIR require resonator dimensions in the hundreds of nanometres range, which can be conveniently fabricated using today’s top-down and bottom-up nanomanufacturing [13,14] as well as wet chemical synthesis techniques [15]. Hence, nanophotonic sensors are strong candidates to become essential elements of modern sensor platforms.

**Figure 1:**
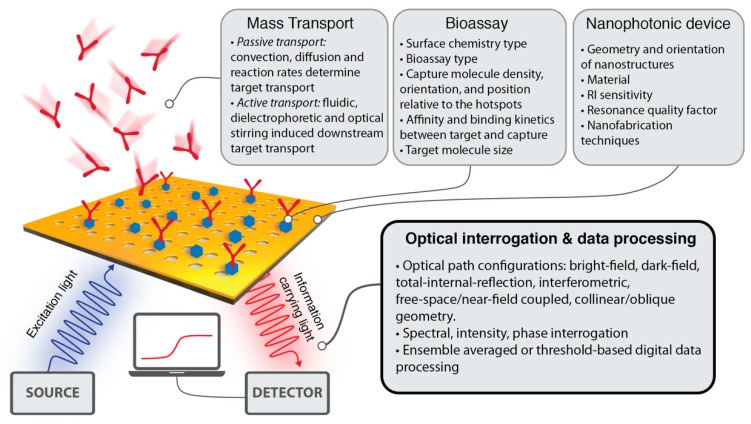
Summary of crucial physical, biochemical, and computational factors affecting the analytical performance of a nanophotonic biochemical sensor.

The majority of the research articles presenting new nanophotonic sensor designs report critical parameters, such as detection limit, dynamic range, and total measurement time, relating these values directly to the optical device performance. However, there are numerous critical factors that affect the overall bioanalytical performance of a nanophotonic sensor platform, as summarized in Figure 1. First, the mass transport technique determines how fast and efficiently the target analytes are delivered onto the sensor surface from the bulk sample volume. Notoriously, the analyte delivery method affects very crucial sensor parameters, such as total measurement time, minimum necessary sample volume to bring the sensor to a dynamic equilibrium, and the minimum amount of analyte that can be detected in a sample. Recently, Spackova et al. [16] reported a universal model considering both the optical and mass transport aspects in nanoplasmonic sensors, highlighting the importance of surface density of capture sites and their proximity to the plasmonic hotspots. In the comprehensive article by Squires et al. [17], the effects of convection, diffusion and reaction rates on target molecule transport is studied in detail based on sensor dimensions and fluidic delivery parameters. In order to shorten the equilibration time and enhance the interaction of target molecules with the surface capture agents, active mass transport methods, such as downstream fluidic flow [18,19], dielectrophoretic stirring [20], superhydrophobic delivery [21] and optical tweezing [22,23,24] have also been proposed.

The second major contributor to the overall performance of the nanophotonic sensors is the binding efficiency of target analytes to the surface-anchored molecular probes. Here, the primary objective is the robust immobilization of the capture molecules to the most sensitive hotspots of inorganic nanoresonators with appropriate orientation of their active binding sides to maximize biorecognition capabilities [25,26,27]. For plasmonic sensors, robust thiol-based Au functionalization methods have been established [28]. Recently, the compatibility of dielectric metasurfaces with biorecognition assays were achieved through the well-known chemical interaction between silane and surface-bound hydroxyl groups [29,30]. Moreover, the affinity and binding kinetics between target and capture molecules, as well as target molecule size and bioassay type, play important roles in the sensor performance as they determine the total mass accumulation on the sensor at equilibrium.

At the core of the nanophotonic sensors’ performance lies the optical resonance properties of these devices including near-surface RI sensitivity and resonance Q-factor. Previously published reviews on nanophotonic biosensors extensively covered the broad range of nanoresonator designs, including different geometries, and materials discussing the physics behind the optical resonance phenomena, as well as their nanomanufacturing techniques [12,31,32,33,34,35,36,37,38,39,40]. 

In this review article, you will find an overview of far-field optical interrogation methods and their associated data processing techniques that have been used to extract detection information from nanophotonic devices, including both nanoplasmonic and dielectric metasurfaces. Uniquely, light has numerous physical aspects, namely, amplitude (intensity), phase, frequency, and polarization. As light interacts with matter, one or more of these physical facets change, which can be measured using different optical schemes. In fact, since the fundamental physical parameter of light that can be directly measured is intensity, different optical interrogation systems utilize a cohort of light sources and optical tools to convert the changes in phase, frequency and polarization into detectable intensity variations. In particular, the optical techniques overviewed in this article measure the physical parameters of light that are modulated by nanoresonators to extract sensing information.

This review focuses on three fundamental interrogation methods: spectral (Section 2), intensity (Section 3) and phase (Section 4), and ends with a brief discussion on integrated nanophotonic devices (Section 5) followed by an outlook (Section 6). It is important to note that the performance of these optical interrogation methods depends on the specifications of the optical tools, such as photon detectors and light sources, which advance in time with the technological developments. Herein, these optical readout schemes are evaluated in terms of their capabilities to satisfy the requirements of next-generation sensors dedicated to various applications. Namely, the size, cost, robustness, capability to perform multiplexed detection, spatial and temporal resolution, and sensitivity of previously reported optical interrogation methods are considered.

## 2. Spectral Interrogation

**Figure 2:**
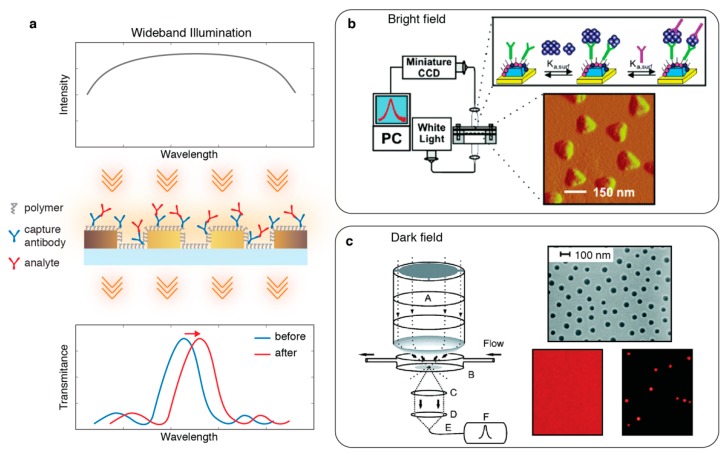
(**a**) Spectral interrogation of nanophotonic sensors. In this representative schematic a collinear light-path is shown, where broadband illumination excites the localized surface plasmons creating a resonance peak in transmission spectrum. The red-shift in the spectral position of the resonance peak can be monitored to extract quantitative analyte binding information; (**b**) Single-point spectroscopy in a bright-field transmission configuration was used to interrogate a nanoplasmonic sensor for the detection of an Alzheimer biomarker. Reprinted with permission from [48]. Copyright 2005 American Chemical Society; (**c**) Single-point spectroscopy in a dark-field transmission configuration was used to interrogate short-range ordered nanoholes. Adapted with permission from [50]. Copyright 2005 American Chemical Society.

Most traditional optical settings that interrogate nanophotonic sensors are based on the characterization of frequency components of light transmitted through (extinction spectrum = absorption + scattering) or reflected (scattering spectrum) from the nanostructured surfaces. Commonly, spectrometers are used to measure frequency dependent intensity, which reveals the linear and non-linear [41] optical resonance characteristics. By acquiring time-dependent spectral data, the resonance properties can be monitored in real time as the local dielectric environment changes due to the analyte binding to the sensitive hot-spots as illustrated in the representative schematic in Figure 2a. These temporal measurements allow for the acquisition of dynamic resonance shift spectrograms, which can be used to measure affinity and association/dissociation rates of kinetic binding reactions. Traditionally, this method is used to characterize a given sensor’s performance parameters, such as sensitivity and limit of detection (LoD), based on titration experiments. For this, the resonance shift values recorded at the dynamic equilibrium are correlated with the known analyte concentrations to establish a calibration curve, the slope of which represents the sensitivity. The minimum detectable concentration, i.e., LoD, is then extracted from the intersection of this calibration curve with three times the standard deviation value measured from the temporal spectral fluctuations at the equilibrium. End-point measurements are also commonly employed for quantitative investigations by simply comparing the spectral data acquired before and after the analyte insertion. In the continuous-time spectral acquisition experiments, a vast number of spectra is collected during the analyte binding period. When this temporally and spectrally rich dataset is processed, noise can be reduced through data averaging, and gradual signal variations, such as drift due to bioassay artefacts, can be digitally corrected. Therefore, this method usually enables measurements that are more robust against bioassay-induced perturbations, particularly for the detection at low analyte concentrations.

The temporal and end-point spectral acquisition techniques can be performed either with in-flow assays, where analyte solution is continuously inserted through a microfluidic channel, or with static assays in which the sample is loaded and incubated in a measurement chamber. The former allows for a more efficient analyte transfer to the surface due to convection, and therefore the dynamic equilibrium that leads to signal saturation can be reached more rapidly. Contrary to the static assays, in-flow experiments require microfluidic sample delivery systems and mechanical pumps, which may lead to more complex sensor instrumentation and introduce noise to the system. Thus, the choice of assay type should be determined based on the application needs.

Spectral information from nanophotonic sensors can be collected using various optical configurations, here, they are categorized under three different groups based on their spatial sensor probing characteristics. Under these three groups, numerous illumination schemes, such as bright-field, dark-field and total internal reflection, are discussed in the subsections that follow.

### 2.1. Zero-Dimensional (0D): Single Point Spectroscopy

In single point spectroscopy, the information carrying light, either transmitted or scattered from a particular region of the nanostructured surface, is evaluated. Due to its simplicity, the most common optical configuration uses bright-field settings, where a broadband light beam illuminates the sensor surface and the light transmitted through the nanophotonic device is collected and coupled to an optical fiber as shown in Figure 2b. Fiber optically coupled spectrometers measure the intensity of the grading-dispersed light beam at different angular positions, using a variety of photon detectors, such as charge-coupled device (CCD), CMOS, and photodiode arrays, depending on the application and spectral range. These modular instruments are usually compact and accessible; therefore, they have been widely used as optical readers for nanophotonic sensors. The measured single-channel extinction spectrum represents the average spectrum of a sensor region, whose area depends on the field of view of the objective. Since this method does not record spatial information, in this review, it is referred as zero-dimensional (0D) single-point spectroscopy.

After the demonstration of single-point spectroscopic interrogation on a collinear transmission path for LSPR sensing [42], a large scope of successful implementations followed including virus detection [43], bacteria growth monitoring [44], exosome profiling for cancer diagnostics [45], quantification of lipid membrane associated species [46], liposome deformation profiling [47] and detection of Alzheimer biomarker from clinical cerebrospinal fluid samples [48]. The first biosensing attempts using dielectric metasurfaces were also reported based on single-point spectroscopy [30,49]. 

In the dark-field configuration, short-range ordered nanoholes were characterized using single-point spectroscopy as shown in Figure 2c [50]. Here, white light is tightly focused on the sensor surface through a high numerical aperture (NA) dark-field condenser, and the scattered light is collected with a high-NA objective and fiber-guided to the spectrometer.

Due to its mature and small-footprint technology, fiber-coupled spectrometers allow for portable, robust and low-cost optical readers that can be built with off-the-shelf optical elements. Yet, this optical interrogation technique suffers from the lack of spatial resolution and throughput, impeding multiplexed signal acquisition. In order to surpass these limitations, mechanical scanning systems were proposed to measure multiple analytes from different sensor regions sequentially [51,52]. The second major drawback of this interrogation technique is spatial ensemble averaging. For low-analyte concentrations, where the surface molecule binding density is low, the spectral shift generated by the analyte binding is averaged with the rest of the bare sensor area. Thus, the miniscule spectral changes generated by individual target analytes that bind on the sensitive hotspots are shadowed by the background noise. Moreover, in the dark-field scattering spectroscopy of NPs, the ensemble averaged spectrum of randomly positioned NPs with inherent size variations generates broader resonances, which is detrimental for sensing [53]. In fact, spatial signal averaging is one of the major hindrances in nanophotonic sensing.

### 2.2. One-Dimensional (1D): Imaging Spectroscopy

**Figure 3:**
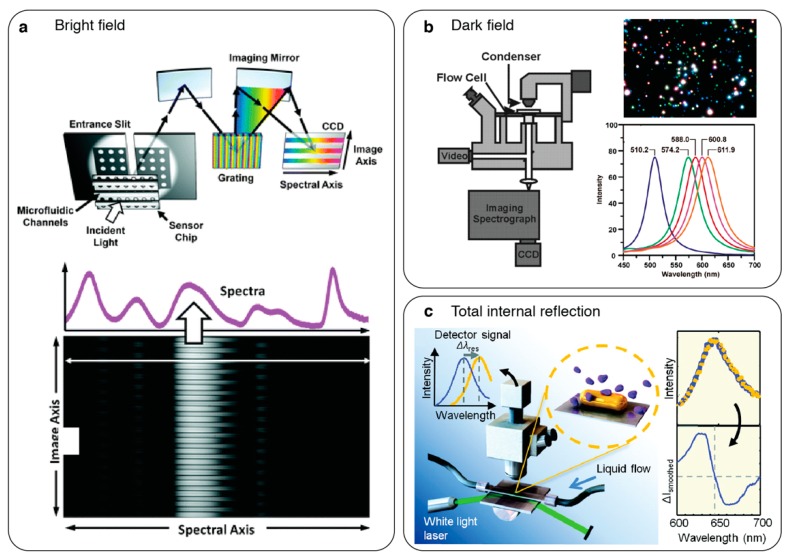
Imaging spectroscopy-based one-dimensional (1D) spatial optical interrogation of nanophotonic sensors. (**a**) (top) Bright-field transmission configuration used to interrogate plasmonic Ag nanohole arrays for real-time multiplexed affinity measurements. (bottom) An exemplary image showing spectra corresponding to the sensor area imaged through the entrance slit. Reproduced from [55] with permission from The Royal Society of Chemistry; (**b**) (left) Dark-field transmission configuration used to interrogate single Ag nanoparticles in real-time with zeptomole sensitivity. (right) Dark field image of nanoparticles and their spectra showing resonance peak shift as the top media refractive index changes. Reproduced from [61] and [58]. Copyright 2003 Society of Photo-Optical Instrumentation Engineers (SPIE) and Copyright 2005 American Chemical Society; (**c**) Total internal reflection setup to interrogate individual plasmonic nanoparticles for time-resolved single protein detection with minimal background interference. Spectral changes in the resonance peak showing the molecule binding events. Adapted with permission from [63]. Copyright 2012 American Chemical Society.

Advancements in imaging technology led to the development of imaging spectrometry, which enables access to the full-spectral information from one-dimensional (1D) sensor regions. In these optical systems, the information carrying light beam passes through a narrow slit, after which it is dispersed by a grating and projected onto a 2D imager, such as a thermally cooled CCD, generating an image that consists of spatially dependent spectral information (see Figure 3a). The implementation of imaging spectroscopy in a bright-field setting for label-free chemical sensing was introduced by Steward et al. [54] using nanoplasmonic devices. On a similar optical setting, the high-throughput sensing was successfully shown by Lee et al. [55] using a multichannel microfluidic network assembled perpendicular to the slit direction. In this work, large-area and spatially coherent plasmonic Ag nanohole array (NHA) chips along with a 50-channel microfluidic flow-cell were used to acquire multiplexed spectral data from each channel in real-time. Imaging spectrometry was also used to interrogate plasmonic Au-NHA sensors for different sensing applications, such as time-resolved analysis of live cell secretion [56] and multiplexed detection of different bacteria species from urine samples [57].

Imaging spectrometers have been particularly beneficial for dark-field spectrometry to measure the LSPR characteristics of metal-NPs dispersed randomly on a substrate (see Figure 3b) [53,58,59,60,61,62,63]. Such systems allow for the acquisition of scattering spectra from individual NPs generating sharp and spectrally isolated resonance curves, which are crucial for LSPR sensing [53,60]. Mayer et al. [60] individually monitored the spectra of Au bipyramid particles and achieved the detection of single molecule binding/unbinding events in real-time. This was a significant achievement because it allowed for the study of label-free stochastic single molecule binding kinetics, which is of great interest in many fields, including proteomics, pharmaceutical drug development and diagnostics. Scattering spectra of metal NPs were also measured using total internal reflection (TIR) settings, in which the particles were excited by an evanescent field created by TIR of broadband light in a glass prism and the NP scattered light is collected by an objective and analysed by an imaging spectrometer (see Figure 3c) [63,64]. Ament et al. [63] used this method to not only show single molecule detection, but also to study the binding kinetics and conformational protein dynamics of individual molecules by analysing the temporal changes in the resonance properties of single Au-NPs. 

### 2.3. Two-Dimensional (2D): Hyperspectral Imaging

Hyperspectral imaging (HSI) techniques enable spatially resolved spectral data acquisition for parallelized spectral interrogation from large regions of a sensor. Specifically, this optical methodology generates three-dimensional (3D) data sets (also called data cubes) comprising 2D spatial images, which retain intensity values from a sensor region measured at different wavelengths. HSI can be implemented using various optical configurations, including wavelength-scan and spatial-scan methods [65]. In the former, tunable light sources or variable-filters are used to control the wavelength of the light at which individual images are recorded, therefore, spectral resolution of this measurement configuration depends on the bandwidth of the tunable light source or the filter as well as the tuning step-size. The spatial resolution of wavelength scanning systems depends on the wavelength, NA of the objectives used in the light path, and pixel size of the imaging device, and it can be as low as diffraction limited spot as in any imaging system. In the spatial-scanning method, either single-point or imaging spectrometers can be employed to acquire spectrum from partial locations of the sensor sequentially to map the sensor spectral properties [66,67,68]. With this method, spectral resolution is defined by the spectrometer parameters, while the spatial resolution is limited by the precision of the mechanical scanning tools. 

**Figure 4:**
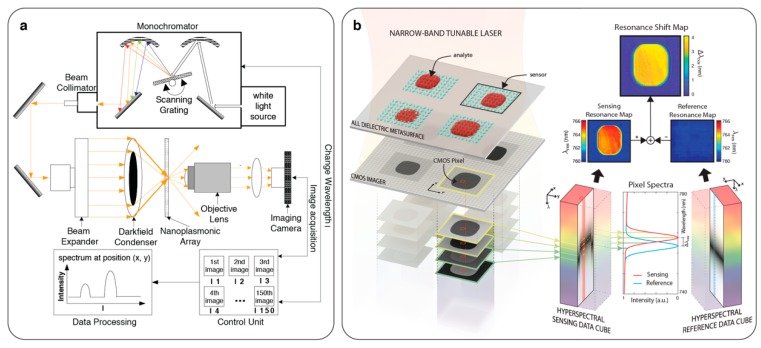
Hyperspectral imaging-based two-dimensional (2D) spatial optical interrogation of nanophotonic sensors. (**a**) Dark-field transmission configuration used to acquire scattering spectra from many individual plasmonic nanoparticles simultaneously. Adapted with permission from [71]. Copyright 2005 American Chemical Society; (**b**) bright-field transmission configuration used for high throughput spatial and spectral data acquisition from high-Q dielectric metasurfaces. Adapted with permission from [29]. Copyright 2019 Nature Publishing Group.

The HSI technique was previously exploited to interrogate LSPR based nanoplasmonic sensors using variable-filters to achieve parallelized measurements [69,70]. Ruemmele et al. [69] introduced a proof-of-concept implementation of this method in a bright-field transmission setting using a liquid crystal tuneable filter and broadband excitation. In this work, metal NP-arrays were spectrally characterized and multiplexed detection was shown by quantifying the resonance shift in the extinction spectrum. Moreover, dark-field configuration of HSI has been employed for single-particle LSPR sensing, as it allows for the scattering spectrum identification of individual metal-NPs randomly distributed on a substrate (see Figure 4a) [66,67,71,72,73,74]. Using this method, Wang et al. [66] presented quantitative epigenetic screening of single cells by localizing two types of NPs (Au and Ag) based on their scattering spectra. In this work, each particle type is labelled specific to a particular epigenetic mark, and analysis of the HSI data cube provided both spatial and quantitative information from individual NPs enabling multiplexed measurement.

Recently, HSI was utilized to collect high-throughput spatial and spectral data from dielectric metasurfaces with high-Q resonances in the NIR [29]. Contrary to the conventional spatial ensemble averaging method, in this work, thresholding-based digital data processing from discrete pixel spectra was employed. Remarkably, the digital data processing approach together with the sharp optical resonances pushed the detection limit down to unprecedented a few molecules per micron square levels. Different than previous HSI optical configurations, in this work, high spectral resolution was achieved by coupling a supercontinuum laser output to a laser line tunable filter, which provides extremely narrow bandwidth (~2 nm) excitation with 0.1 nm tuning steps. Due to the high spectral power density in VIS-NIR wavelengths of the laser source, large sensor areas could be illuminated using narrowband source without light-starvation enabling the acquisition of large field-of-view (FOV) images (see Figure 4b). The strong potential of HSI based optical interrogation for high throughput biosensing was also shown by Juan-Colas et al. [75] for parallel, in vitro, and real time single-cell secretion analysis using 2D photonic crystals.

### 2.4. Spectrometer-Less Spectral Interrogation

**Figure 5:**
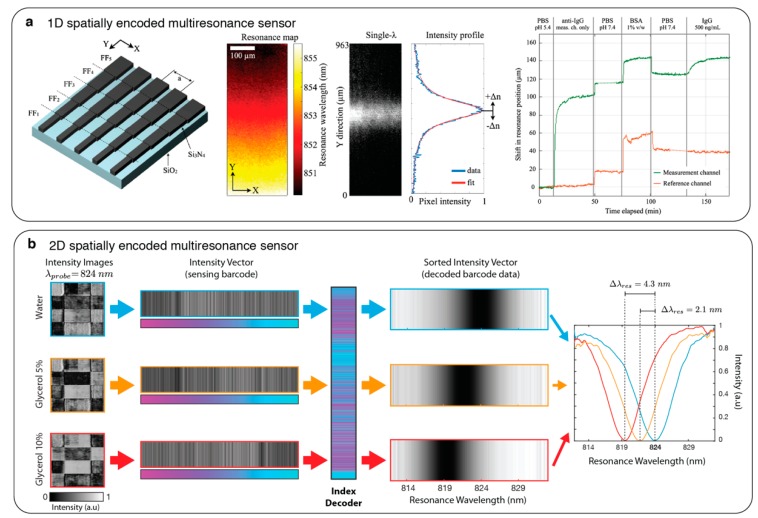
Spatially encoded spectral interrogation with multi-resonance nanophotonic sensors. (**a**) (left) Chirped guided-mode resonant grating where spectral resonance position is encoded in Y-axis as shown in the resonance map. (middle) Bright-field image of the grading and its corresponding intensity profile when illuminated with monochromatic light. (right) Temporal resonance position plot in an in-flow bioassay. Adapted with permission from [76]. © 2017 Optical Society of America; (**b**) barcode-based sensing encoding the high-Q resonance positions of dielectric metasurfaces into 2D multiresonance sensors. (Left to right) Monochromatic intensity images of a multiresonance sensor in the presence of different top-media, their corresponding sensing barcodes, index decoder, decoded barcodes and the spectra showing the resonance shifts in accordance with the top media refractive index. Adapted with permission from [29]. Copyright 2019 Nature Publishing Group.

Spectrometers have been the most commonly used instruments to analyse the optical resonance properties of the nanophotonic devices enabling their extended use as sensors. However, their cost, dimensions and technological complexity strictly defines spectrometers’ technical specifications, which closely impact the sensing performance. Therefore, relying on spectrometers hinders the development of miniaturized and low-cost systems. Recently, two independent groups presented spectral interrogation of nanophotonic sensors without using spectrometers and dispersive optics [29,76]. Both techniques utilized multiresonance nanophotonic devices, which were designed by spatially encoding geometrically tuned nanoresonators to continuously cover a defined spectral range. Thus, simply from a simple image of these multiresonance sensors illuminated by a narrow band light, it was possible to probe the RI changes in the top media. Triggs et al. [76] employed chirped guided-mode resonances and tuned their resonance position by gradually changing the grading width along one dimension creating a resonance map, which changes continuously in one dimension as shown in Figure 5a. Imaging this sensor with a narrow band light generates a spectrum-like intensity profile as each axis position modulates the light transmission based on its resonance position. Interrogating the intensity peak shift with respect to the axis position, the changes in the RI of the top media was demonstrated. In the second approach, Yesilkoy et al. [29] achieved spatial encoding of the optical resonances by tuning the metaunit dimensions in a dielectric metasurface. Contrary to the first approach, here any CMOS pixel in the image can be used to probe a specific spectral position determined by high-Q metaunit resonances inspired by the physics of bound-states-in-the continuum (BIC). Decoding the intensity values from a single image using the fingerprints of the multiresonance sensors, spectral information was extracted and RI sensing was achieved (see Figure 5b). These new methodologies are promising as they allow for robust spectral interrogation using simple optical tools offering great prospects for miniaturization.

## 3. Intensity Interrogation

**Figure 6:**
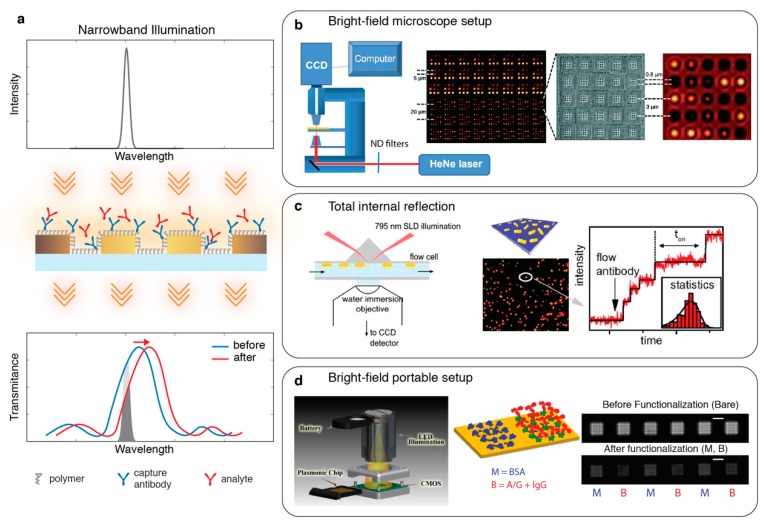
(**a**) Intensity interrogation of nanophotonic sensors. In this representative schematic a collinear light-path is shown, where narrowband illumination is tuned to a flank of the resonance peak in transmission. The red-shift in the spectral position of the resonance peak induces a change in transmission, which can be monitored to extract quantitative analyte binding information; (**b**) (left) Bright-field transmission configuration used for high throughput intensity interrogation. (middle to right) Bright-field image of a large microarray of sensing pixels, which are composed of a set of nanohole arrays with varying periodicity surrounded by Bragg mirrors, scanning electron microscope (SEM) image of the sensor, bright-field image of a single sensing pixel. Reproduced with permission from [77,78] Copyright 2008 Optical Society of America and Copyright 2009 The Royal Society of Chemistry; (**c**) (left) total internal reflection configuration used to monitor stochastic protein interactions from hundreds of single-molecule plasmonic sensors. (right) Time-traced scattering intensity of individual nanoparticles, stepwise intensity changes show stochastic single antibody binding events. Adapted with permission from [83]. Copyright 2015 American Chemical Society; (**d**) (left) schematic of a portable biosensing device used for computational imaging of Au nanohole arrays. (right) Formation of protein layers result in intensity decrease as shown in the bright-field images. Adapted with permission from [84] Copyright 2014 Nature Publishing Group.

Multiplexed detection on miniaturized sensor platforms is a critical expectation from next-generation sensors. This urgent need motivated the implementation of intensity interrogating optical readers to simply measure the changes in the optical resonances of nanoplasmonic sensors using imaging detectors, such as CCD or CMOS cameras and low-cost modular light sources. Specifically, a narrowband light, which is spectrally aligned to the sharp side slope of the resonance, is used for excitation. The camera pixels monitor the intensity variations that are correlated with the spectral shifts induced by the local RI changes on the sensor regions (see Figure 6a). Intensity interrogation was demonstrated to excite plasmonic NHAs by Lesuffleur et al. [77] using a laser light source and a CCD camera assembled on the collinear light path of an inverted microscope for bright-field imaging (see Figure 6b). In order to increase the lateral resolution and block the crosstalk between the sensor arrays, Lindquist et al. [78] added Bragg mirrors around the NHA sensors, which led to small-sensor units and increased the throughput in a single measurement (see Figure 6b). Later this technique was further explored by various groups enhancing its sensitivity, throughput and showing real-time multiplexed detection of biomolecules using different nanoplasmonic transducers [79,80,81,82].

Intensity interrogation was also implemented using dark-field imaging optics for two types of studies. In the first approach, Beuwer et al. [83] used high-NA objective to monitor time-resolved intensity variations from individual NPs due to the spectral shifts induced by the molecular binding events. In this work, a narrow bandwidth super-luminescent diode was used for illumination in a TIR geometry and the NP-scattered light was collected by an objective and imaged by a CCD camera with minimal background interference (see Figure 6c). Monitoring intensity variations from hundreds of NPs simultaneously, high-throughput data acquisition was achieved. This allowed for the statistical analysis of single molecule binding kinetics contrary to the ensemble averaged data provided by conventional techniques. In the second approach, ensemble intensity variations from microarrays of NPs were measured from dark-field images using low-NA objectives with larger FOV. This method was used for multiplexed detection of serum cytokines for diagnostic purposes [85], temporal profiling of T-cell secretion [86], and to monitor the secretion from tissue-on-chip platform [87].

In a parallel effort for miniaturization, a hand-held sensing platform was demonstrated by Cetin et al. [84] where a lens-free light path was used together with computational imaging to enable quantitative biomolecule detection using nanohole sensor arrays (see Figure 6d). Later, to optimize the performance of a similar platform, machine learning algorithms were employed for smart selection of excitation LED sources [88]. A portable intensity interrogating plasmonic nanohole sensor was also proposed for the detection of uropathogenic *E.coli* by Gomez-Cruz et al. [89] for the point-of-care urinary tract infection diagnostics.

**Figure 7:**
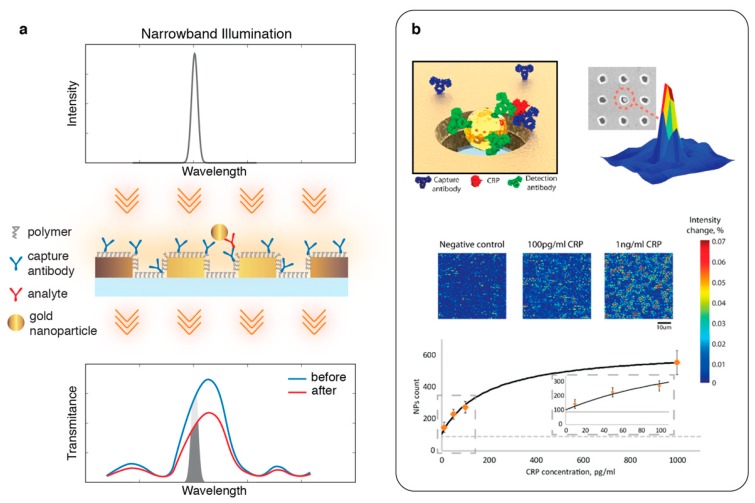
(**a**) Nanoparticle-enhanced plasmonic imaging. Schematic shows a collinear transmission light-path, where a narrowband illumination tuned to the flank of the Au nanohole array resonance peak is used for bright-field imaging. Nanoparticle binding at the nanoholes distort the localized plasmons creating a drastic suppression of the transmission peak, which can be monitored to extract digital analyte binding information; (**b**) (top) schematic shows a sandwich bioassay, antigen being recognized by capture antibodies immobilized on the Au-nanoholes and then by detection antibodies tethered to Au-nanoparticles. Strong local suppression in the transmission create intensity contrast. (bottom) Bright-field images and a calibration curve for human C-reactive Protein detection. Adapted with permission from [90] Copyright 2018 American Chemical Society.

Despite the crucial benefits offered by the intensity interrogation techniques applied to nanophotonic sensors, the trade-off between achieving clinically relevant sensitivity levels and high-throughput detection using simple, inexpensive optics remained. In order to address this, Belushkin et al. [90] proposed a NP-enhanced wide-field imaging based plasmonic biosensing technique. In this detection scheme, analyte molecules were detected in a sandwich assay where capture and detection antibodies were conjugated onto the Au-NHA sensor surface and the Au-NPs. Thus, Au-NPs become attached onto Au-NHA surface specifically through the analyte binding to the antibodies. Distorting the localized plasmons, individual sub-wavelength Au-NPs suppress the plasmonic resonance peak creating a significant intensity contrast, which can simply be detected using low-NA objectives and inexpensive CMOS imagers (see Figure 7a). Quantifying the high contrast spots from the plasmonic images shown in Figure 7b, the individual NP-labelled analyte molecules could be detected over large sensor areas. This digital biomolecule sensor enables the multiplexed detection of biomarkers at low concentrations (LoD of 27 pg/mL for C-reactive protein) similar to the current gold-standard clinical laboratory techniques, such as ELISA.

## 4. Phase Interrogation

**Figure 8:**
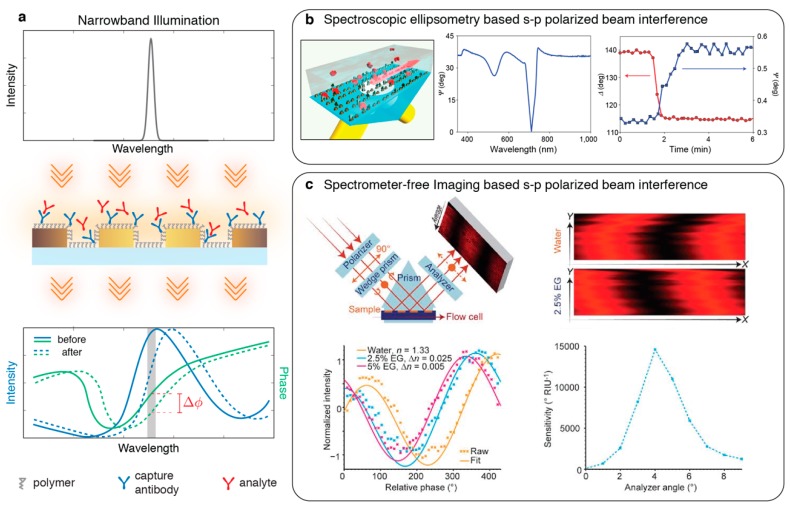
(**a**) Phase interrogation of nanophotonic sensors. In this representative schematic a collinear light-path is shown. A narrowband light tuned to the localized surface plasmon peak, where the phase shift gradient is high, is used for excitation. The red-shift in the spectral position of the resonance leads to a shift in the phase function, which can be interrogated using an interferometry technique to extract quantitative analyte binding information; (**b**) (left) singular phase response of plasmonic metasurface was probed by spectroscopic ellipsometry in a total internal reflection configuration. (middle) Ellipsometric spectral response of nanoplasmonic sensor. (right) Time-traced phase change signal measured during a bioassay. Adapted by permission from Nature Publishing Group [91], Copyright 2013; (**c**) (top-left) Schematic showing reflection optical configuration where the nanoparticle layer was interrogated using spatially varying polarization states of a collimated laser diode beam. Interaction between the p- and s-polarized reflection components create interference fringes on a charge-coupled device (CCD) imager (top-right). (bottom-left) The fringes shift as the ambient refractive index increases. (bottom-right) Sensitivity dependence on the analyzer angle. Adapted with permission from [92] Copyright 2014 Nature Publishing Group.

Another physical facet of nanophotonic resonance phenomenon reveals itself as an abrupt phase change in the spectrum due to the temporal retardations of resonantly coupled electromagnetic waves to the surface plasmons with respect to the uncoupled propagating background. The gradient of the spectral phase response peaks at the center resonant wavelength and depends on the quality factor of the resonance. Probing the phase jumps instead of intensity peaks or dips corresponding to the plasmonic resonances for sensing offers a great potential to improve sensitivity (see Figure 8a). Phase interrogation requires interference of an information carrying light with an unaffected reference beam to convert the phase changes into physically detectable intensity signals. The key benefit of phase interrogation becomes evident when common path interferometry techniques are used, where the reference and the signal beams are accessed through the same optical path, therefore are affected by the same noise components. Consequently, phase interrogation can minimize the background and non-specific environmental noise as the measured signal is always referenced.

While the non-trivial requirements of optical interferometers obstruct the widespread exploration of phase interrogation techniques, numerous techniques have been proposed showing improvements in certain performance parameters of nanoplasmonic sensors.

### 4.1. Polarization Interferometry

Kravets et al. [91] implemented the common path phase interrogation using spectroscopic ellipsometry, where the localized plasmon resonances in an array of Au double-nanodots were excited by diffractive coupling (see Figure 8b). In this optical setting, p-polarized component of a broad band light beam, which excites the surface plasmons and carries the resonance change information, was referenced with the unaffected s-polarized component. The s- and p- polarized components were interfered with and spectrally analysed to measure the phase variations for sensing. Alternatively, polarization interferometry was implemented by Otto et al. [93] in a collinear reflection geometry using an inverted microscope’s light-path. To extract the spectral phase shift data, a liquid crystal variable plate was employed. Yet, similar to 0-D spectroscopy, these optical interrogation techniques can only probe a single point on the sensor, thus cannot provide high-throughput spatial information. Moreover, spectroscopic polarization interferometry instruments are bulky and expensive tools, and do not satisfy the portability and low-cost instrumentation demands of future sensors. Svedendahn et al. [92] proposed a spectrometer-free phase interrogator as a low-cost alternative to ellipsometric spectrometers. In this optical scheme, a single-wavelength excitation source was spectrally tuned to a wavelength at which a sharp phase change occurs and a CCD camera was used to detect interfering s- and p- polarized beam components (see Figure 8c). Here, a gradually varying phase delay along one spatial axis is introduced using a wedge prism and the phase variations on the p-polarized component due to the resonance shift is encoded as a fringe pattern drift along one axis. By utilizing one spatial dimension in this imaging-based technique, the need of spectrometers to monitor the phase shift was eliminated.

**Figure 9:**
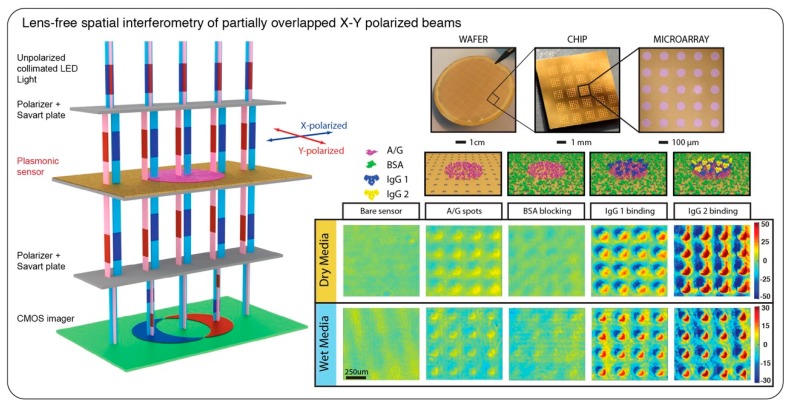
(left) Large field-of view common-path spatial interferometry. In the collinear optical light-path collimated light-emitting diode (LED) light beam is first linearly polarized at 45° and then x- and y-polarized components are sheared by a savart plate (a birefringence element). This generates quasi-spatially overlapped and orthogonally polarized light beams that traverse the plasmonic microarray plate and are subsequently recombined using a second savart plate and interfered by a second polarizer. The interferogram, showing the fringes, is finally imaged by the complementary metal oxide semiconductor (CMOS) sensor. (right-top) Wafer-scale fabricated Au nanohole arrays cover the whole chip uniformly, allowing for high-throughput sensing. (right-bottom) Protein microarray detection on a plasmonic phase interrogation scheme. Adapted with permission from [94] Copyright 2018 Nature Publishing Group.

In the work by Yesilkoy et al. [94], a large field-of-view lens-free spatial interference contrast technique [95] was used to exploit the sharp phase transitions of plasmonic Au-NHAs for high-throughput sensing. In this phase interrogating scheme, x- and y- polarized components of a collimated light-emitting diode (LED) beam was spatially sheared in a collinear transmission geometry and after traversing the plasmonic sensor chip were remerged and interfered. On the CMOS imager, the fringe patterns corresponding to the phase contrast between the specifically patterned sensing micro-spots and the sensor background were detected as a result of their spatial interference (see Figure 9). This optical platform was built with off-the-shelf electronic and optical products as a portable and low-cost reader for point-of-care biosensing applications. Moreover, the plasmonic chips were produced using scalable nanofabrication techniques over large areas enabling the simultaneous detection of thousands of protein biomarker spots on a compact and robust microarray reader setting as shown in Figure 9. This optical interrogation technique enabled an order of magnitude improvement in the detection limit of proteins [94], allowing for early detection of sepsis biomarkers [96]. Furthermore, using this technique, bacteria detection was demonstrated from human blood plasma samples distinguishing sepsis patients from healthy controls [97].

### 4.2. On-Chip Surface Plasmon Interferometry

**Figure 10:**
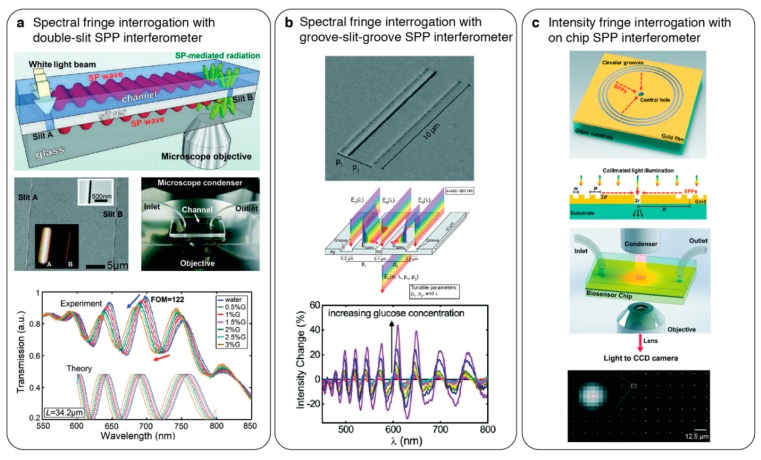
On-chip surface plasmon polariton interferometry. (**a**) (top) Schematic showing an on-chip Mach–Zehnder interferometry configuration. Nanoslit coupled propagating plasmons on the sensing channel and reference substrate interfere at the second slit where an objective collects the scattered light and fringe analysis is performed by a spectrometer. (middle) SEM showing the nanoslits and photograph of integrated microfluidic chip. (bottom) Spectra showing fringe shift as the refractive index of the sensing media increase. Adapted with permission from [98] Copyright 2011 American Chemical Society; (**b**) (top) SEM of a groove-slit-groove planar plasmonic interferometer where the groove distances to the slit are uneven. (middle) Schematic showing the working principle of plasmonic interferometer. Diffractively coupled surface plasmons at the grooves counter-propagate and interfere with the free-space propagating light at the slit where the transmitted light carries the sensing information. (bottom) Plot shows intensity changes in the fringe shifts as the top media refractive index increases. Adapted with permission from [99] Copyright 2012 American Chemical Society; (**c**) (top) intensity interrogating surface plasmon interferometry implemented by circular grooves and central hole geometry. (middle) Schematic showing collinear optical path with a monochromatic illumination probing the fringe shifts. (bottom) Bright-field image recorded by a CCD camera showing a large array of interferometers. Reproduced from [100,101] with permissions from The Royal Society of Chemistry.

Phase interrogation of nanoplasmonic sensors was also achieved by directly interfering propagating surface plasmons on a chip without the need of an external beam interfering with the optics [98,99]. Gao et al. [98] employed two parallel sub-wavelength slits in a continuous Ag metal on a glass substrate and excited the plasmons both on the metal-air (sensing) and metal-glass (reference) interfaces using broadband illumination through the first slit (see Figure 10a). The light collected from the second slit encompassed the interference patterns of the propagating plasmons on the sensing and the reference paths. By spectrally analysing the fringe patterns, real-time protein detection was demonstrated. Similarly, on-chip phase interrogation was also implemented using groove-slit-groove structures [99,100]. Herein, the interference of the groove-coupled propagating plasmons probing the analyte binding events on the metal surface were interfered with the reference transmission through the slit (see Figure 10b). In addition to the spectral evaluation, groove-slit interferometry also allows for the intensity interrogation of the fringes using a narrow band light and imaging detectors, enabling high-throughput sensing and inexpensive instrumentation [101] as shown in Figure 10c. 

## 5. Integrated Nanophotonic Sensors

**Figure 11:**
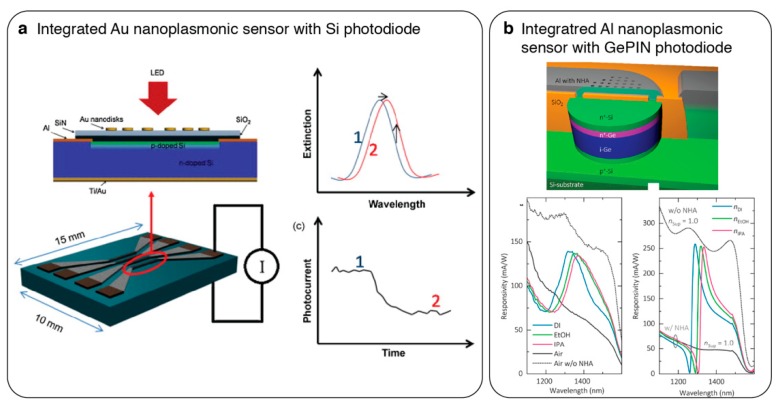
Integrated nanoplasmonic sensor and photon detector systems enabling direct conversion of optical detection signals into electrical signals. (**a**) Plasmonic nanodisks are directly fabricated on Si photodiodes. When illuminated with a LED light source, the change in transmission is measured as photocurrent based on the intensity interrogation technique. Reprinted from [102], Copyright 2010, with permission from Elsevier; (**b**) CMOS compatible Al nanoholes are patterned on Ge PIN photodiode. Adapted with permission from Ref. [107] Copyright 2011 American Chemical Society.

Monolithic integration of nanostructured optical sensors with semiconductor photon detectors is a promising approach for miniaturization as it eliminates the use of external spectrometers and cameras, thus reducing the total footprint [102,103,104,105,106,107]. Frequently, these integrated on-chip sensors are illuminated with an external narrow band light source in a collinear path. Nanoplasmonic structures patterned directly on top of photon detector arrays modulate the incident light and the transmitted light is converted into electrical signals on the same device (see Figure 11a). Thus far, various nanoplasmonic structures, including nanodiscs [102,106], nanoholes [103,107] and nanogratings [104,105], have been integrated with numerous photon detectors, such as Si/Ge photodiodes and metal oxide semiconductor (MOS)/CMOS detectors, for intensity interrogation. In order to enhance the sensitivity of an integrated nanoplasmonic system, Guyot et al. employed Au NHAs with asymmetric resonance properties. In this self-refencing intensity interrogation technique, the NHAs have different lattice properties in X and Y directions, therefore, their spectral resonances vary when illuminated with X and Y polarized light. When the system is excited with a laser source spectrally aligned to the intersection of these two resonances, the intensity difference between the X and Y polarized transmission is enhanced.

To fully benefit from the integration of nanoplasmonic sensors with other photonic elements, it is key that they are fabricated using compatible mass-manufacturing techniques. To address this challenge, Augel et al. [107] proposed the integration of plasmonic Al NHAs with Ge PIN diodes as cost-effective ultracompact on-chip biosensors (see Figure 11b). Contrary to previous reports, the materials used in this work were fully compatible with CMOS manufacturing, opening the way for low-cost mass-fabrication of such sensors. 

## 6. Outlook 

In the past two decades, there has been a tremendous progress in the nanophotonic biosensor development, enabled by substantial multidisciplinary research efforts in the fields of engineering, physics, chemistry, and life-sciences. An important facilitator has been the commercially available and user-friendly numerical simulation tools, which enabled the optimization of device design by exploring geometrical and material parameters. Moreover, these 3-D solvers advanced the understanding of optical resonance characteristics, elucidating the interaction of nanostructures with light. On the experimental front, the research on the nanophotonic sensors spurred as nanofabrication facilities became accessible for lithographic manufacturing of nanoresonators. In parallel, the availability of straightforward metal-NP synthesis protocols as well as the increase in the number of commercial NP providers significantly contributed to the development of the nanophotonic sensors. Akin to these advancements, the flourishing microfluidic technologies supplemented the development of integrated lab-on-chip biosensing platforms enabling their use in real-world applications. All of these engineering efforts were crowned when robust surface chemistry methods were adapted to interface these inorganic sensor surfaces with biological entities. In this review, I have focused on the last component that allowed for nanophotonic biosensors to flourish: optical techniques used to probe the nanophotonic resonances for sensing, highlighting some of the seminal articles, and discussing the importance of read-out methods on the nanophotonic sensors’ potential to address the needs of next-generation sensing platforms.

Despite the recent massive advancements in the technology development front, nanophotonic biosensors still need to prove their relevance and reliability in real-world applications to be able to challenge the currently established gold-standard sensing methods. One way to do so is to find niche applications with technical requirements that cannot be met by the current standard laboratory techniques. For example, the real-time and label-free characteristics of nanophotonic sensors enable continuous data acquisition with high temporal resolution. These are crucial for experiments that study fast-kinetics of secretion from live cells or tissues, which cannot be achieved with the traditional labelled detection methods with discrete sensing times from bulk supernatant samples. Moreover, with high-throughput optical interrogation methods, such as intensity interrogation and hyperspectral imaging, as recently presented by Juan-Colas et al. [75], high temporal and spatial resolution can be accomplished on the same platform empowering live cell studies that would not be possible otherwise. Furthermore, nanophotonic sensors can play pivotal roles on the successful implementation of complex organ/organoid/disease-on-a-chip platforms due to their dynamic spatio-temporal secretome monitoring potency. Since these applications are laboratory and wet-bench bound, they do not necessarily impose cost-efficiency or portability needs. Therefore, imaging-based spectral interrogation techniques can be appropriate to achieve high sensitivity while acquiring temporally fast signals from multiple sensor areas.

Single-molecule investigation is another specific application where nanophotonic sensors provide unique solutions as they allow for non-destructive, direct and real-time detection of molecules in their natural states. Single-molecule studies are important in understanding many complex biological processes including protein conformational dynamics, and heterogeneous nature of molecular interaction kinetics. Approaches using plasmonic single-particle measurements have been significant for the single-molecule analytics. A good example of this is the intensity based optical interrogation demonstrated by Beuwer et al. [83] that achieved the monitoring of hundreds of NPs and single-molecule events simultaneously in real-time allowing for high-throughput and statistically significant single-molecule studies. 

In order to secure their place in the next-generation healthcare systems, e.g., as essential analytical platforms used for clinical diagnostics and disease monitoring, nanophotonic sensors need to surpass the capabilities of today’s established clinical methods. First, they need to be portable platforms, which can be operated by non-experts with simple instructions. Moreover, these next-generation portable nanophotonic sensors should allow for on-site operation, therefore temperature and vibration robustness are crucial. Second, detecting multiple analytes from low-volume samples in short-turnaround times would give the nanophotonic sensors an unparalleled leverage to be the pivotal health-monitoring nodes. Nevertheless, before surpassing the challenge of detecting small analyte molecules from complex real samples, where the RI contrast generated by molecular binding with respect to the background is miniscule, the nanoplasmonic sensors cannot make the promised impact. Towards this goal, two recent publications emphasize the importance of threshold-based data processing techniques enabling digital molecule detection, contrary to the conventional ensemble averaging. The NP-enhanced plasmonic imaging technique presented by Belushkin et al. [90], not only achieves detection levels that are similar to gold-standard ELISA from real samples, but also uses a simple intensity interrogating optical setting allowing for multiplexing with a low-cost detection scheme. Furthermore, Yesilkoy et al. [29] showed that thresholding methods, powered by high-Q dielectric metasurfaces and hyperspectral imaging, can identify a few molecules per square micron area, which is impossible using ensemble averaging. 

To conclude, this review systematically presents the optical methodologies used thus far for the implementation of nanophotonic devices as biochemical sensors. It highlights the importance of optical interrogation techniques to unlock their key benefits, which can open the way for nanophotonic biochemical sensors to be one of the major sensing technologies of the future.
